# Major bleeding during negative pressure wound/V.A.C.^® ^- therapy for postsurgical deep sternal wound infection - a critical appraisal

**DOI:** 10.1186/1749-8090-6-121

**Published:** 2011-09-29

**Authors:** Jan J van Wingerden, Patrique Segers, Lilian Jekel

**Affiliations:** 1 Department of Plastic- and Reconstructive Surgery, Academic Medical Center, University of Amsterdam, the Netherlands; 2 Department of Cardiothoracic Surgery, Academic Medical Center, University of Amsterdam, the Netherlands; 3 Department of Cardiothoracic Surgery, Medisch Centrum Leeuwarden, Leeuwarden, the Netherlands

**Keywords:** Bleeding emergency, Chest wall, Mediastinitis, Mediastinal infection, Negative pressure, Shock circulatory, Statistics meta-analysis, Sternum, Ventricle right, Wound infection

## Abstract

Negative-pressure wound therapy, commercially known as vacuum-assisted closure (V.A.C.^®^) therapy, has become one of the most popular (and efficacious) interim (prior to flap reconstruction) or definite methods of managing deep sternal wound infection. Complications such as profuse bleeding, which may occur during negative-pressure therapy but not necessarily due to it, are often attributed to a single factor and reported as such. However, despite the wealth of clinical experience internationally available, information regarding certain simple considerations is still lacking. Garnering information on all the factors that could possibly influence the outcome has become more difficult due to a (fortunate) decrease in the incidence of deep sternal wound infection. If more insight is to be gained from fewer clinical cases, then various potentially confounding factors should be fully disclosed before complications can be attributed to the technique itself or improvements to negative-pressure wound therapy for deep sternal wound infection can be accepted as evidence-based and the guidelines for its use adapted. The authors propose the adoption of a simple checklist in such cases.

## 

Serious bleeding during topical negative-pressure wound therapy (NPWT), commercially known as vacuum-assisted closure (V.A.C.^®^) therapy, for deep sternal wound infection (DSWI), is exceedingly rare.

The source of the bleeding is either from the right ventricle (RV) [1-4] or a vessel (aorta or homograft, or coronary bypass graft). Two mechanisms have been linked to serious bleeding and NPWT therapy: infectious erosion [5,6] or, in the case of the RV, a combination of mechanics (displacement of the heart towards or in between the sternal edges [7] and fibrous adherence of the RV to the sternum [1]).

To these two mechanisms Kiessling and colleagues [8] add, in the most recent volume of the *Journal*, penetration by dislodged bone and/or wire fragments as a cause of severe bleeding. Clearly, neither penetration due to dislodgement nor erosion due to incomplete infection control are *caused *by but may occur *during *negative-pressure therapy.

Does negative- pressure, however unequivocally, *contribute *to an increased risk of critical bleeding? A number of arguments can be put forward against this proposition:

1. Critical bleeding during the "open" (dressing) management of DSWI, prior to the introduction of NPWT, has been rare [9] yet is well known. Interestingly, the pathogenesis suggested and lucidly described by Robicsek [10] in 1997 is not different from that currently associated with NPWT.

2. Whereas internationally NPWT has become one of the most popular (and efficacious) interim (prior to flap reconstruction) or definite methods of managing DSWI, the total number of exsanguinations reported is rarer still.

In the last 5 years (2005-2010), the incidence of bleeding during NPWT for DSWI in the Academic Medical Center, Amsterdam was 3.4% (2 from 58). One was a minor bleeding from the RV, which was stopped with a single stitch, on the ward; the other was major from an infected aorta, which had to be taken back to theatre. The incidence at the Medical Center Leeuwarden was 0% in the 39 cases treated for DSWI during the same period.

3. Interface dressings offer dual, mechanical protection: firstly, the RV is protected from both adherence to and friction from the raw sternal edges. Secondly, interface dressings always result in a loss of negative pressure underneath the dressing, as was demonstrated by the frequently overlooked study of Jones and colleagues [11]. The degree of loss depends on the type of interface dressing used. The highest mean decrease in pressure occurred with the use of paraffin-impregnated gauze [11].

Polyamide nets, impregnated with silicone jell interface dressing (e.g. Mepitel^®^) resulted in the smallest decrease in mean pressure. (Mepitel^® ^is thus a silicone, not a paraffin/petrolatum, dressing - this distinction is important because of the differing reactions of the diverse coating materials to body temperature). Either paraffin-impregnated gauze (e.g. Jelonet^®^) or cellulose, acetate fibre coated with a petrolatum emulsion (e.g. Adaptic^®^) is the most common type of interface dressing used worldwide during NPWT therapy for DSWI. The elegant studies of Petzina and colleagues [12] supported these findings, observing a 53 ± 5 mmHg pressure difference between precardiac space and vacuum source when 4 layers of paraffin-impregnated gauze were interposed. Thus, although more clinical studies are required, negative pressure between the layers of protective gauze and the heart is considerably less than generally suspected. If neither the use of or preference for an interface dressing is mentioned in a study, one could be tempted to underestimate its significance.

The highly respected and productive group from Lund, Sweden, would perhaps counter that an interface dressing may not prevent displacement and bulging of the RV into the diastasis between the sternal edges during NPWT. Malmsjö and colleagues [7] base their argument on an MRI animal study. In their (small) study group the bulging was seen in only 2 of 6 animals. Was the displacement a prelude to rupture? Would the bulging not eventually have occurred in certain cases, in any way? Robicsek [10] believed that RV rupture results from the sudden impact (not continuous) of the RV, in the exposed mediastinum, being squeezed against the restraining edges of the sternum by pressures in the lung and pleural cavities exceeding 300 - 600 mmHg when the patient coughs or struggles. Nevertheless, following their line of thought, Lindstedt and colleagues [13], in the same volume of the *Journal*, present a novel solution to prevent the bulging, which they attribute to NPWT: a rigid disk. Though the idea is applaudable, it may not be sufficient.

During NPWT, bacterial load may, contrary to popular belief, remain quantitatively unchanged or increase unabated [14,15], thereby increasing the risk of infectious erosion. A significant (*p *< 0.05) quantitative increase in *Staphylococcus aureus*, the most common pathogen found in postoperative mediastinitis, was observed in the elegant clinical NPWT- study by Mouës and colleagues [16]. The replication behaviour of coagulase-negative staphylococci (CoNS), which are ever more frequently encountered culprits in cases with DSWI, was not reported. Incidentally, in 2 of the 3 reports [3,5,8] of serious bleeding during NPWT for DSWI, the cultures were CoNS positive. In older publications, the associated microorganisms were seldom mentioned. It is of interest that none of the animals studied by the Lund group were, for obvious ethical reasons, infected (and, as far as we could ascertain, none developed bleeding).

Progress of infection will not only increase the risk of serious bleeding [17], but may also result in the unnecessary prolongation and ultimate failure of NPWT [3,18]. This may manifest itself in one of two ways: failure of the progress of healing or as recurrent sternal infection (RSI). Bapat and colleagues [3] found that two-thirds of the patients requiring readmission for further surgery for RSI had been on NPWT as treatment for DSWI for longer than 21 days. Their observation was supported by another recent study [18] where a significant difference (*p = *0.0145) was shown to exist in the mean time of NPWT prior to sternal osteosynthesis between those patients who developed RSI and those who did not.

The unfortunate, common denominator in all these reports is the relatively small numbers of patients. If we are to base the association, if any, between NPWT and severe bleeding on evidence and further our knowledge of the mechanism, it will require a meta-analysis. If more insight is to be gained from fewer clinical cases treated by NPWT, then various potentially confounding factors should be disclosed. The garnering of sufficient information for a meta-analysis will only succeed if *all *possible determinants are not only recorded, but also assiduously reported. Despite the wealth of clinical experience internationally available, information regarding certain simple considerations is still lacking. These considerations include whether the pressure should be adjusted or the choice of interface altered in certain situations, whether it is only once the right ventricle becomes infected that the risk of rupture increases, whether the complication is purely mechanical, how progress in wound healing is measured, and whether the therapy be stopped after a period of time.

For this reason, we propose that those interested in the field (authors and editors alike) make use of a simple checklist prior to the submission of their manuscripts. The checklist should include, at least: the microorganisms involved, the interface dressing used, the negative pressure setting(s), the frequency of changes of dressing and the total period of NPWT (Table 1). Additional factors worth considering inclusion would be: type of anticoagulation used during treatment, site of CABG- graft to the right coronary artery (if performed), right heart failure, level of dependency of patient when haemorrhage occurred (ventilation, dialysis, etc), extent of debridement performed (presence of wires or bone spicula, etc).

**Table 1:** The microorganisms involved, the interface dressing used, the negative pressure setting(s), the frequency of changes of dressing and the total period of NPWT

Microbiology	*Staphylococcus aureus*
	CoNS
	MRSA
	Other
**Interface dressing**	Type
	Layers

**Negative pressure setting(s) **[*mmHg*]	< 75
	75-100
	100-125
	> 125

**Frequency of dressing changes **[*days*]	< 3
	3 - 5
	> 5

**Total period of NPWT **[*weeks*]	< 3
	3 - 5
	> 5

*CoNS*, Coagulase - negative staphylococci; *MRSA*, Methicillin - resistant *Staphylococcus aureus*;

In conclusion, it is undeniable that NPWT has become a valued method and has often simplified the management of DSWI [19]. Recommendations to prevent complications and improve the efficacy of NPWT are commendable. Nevertheless, we feel that large-scale clinical observational studies making available all the information suggested in Table 1 are essential to establish whether a complication can be attributed to the technique. This would allow the guidelines for its use to be adapted and also for further improvements to NPWT for DSWI.

## Competing interests

The authors declare that they have no competing interests.

## Authors' contributions

JJvW, PS and LJ contributed equally to this work. JJvW drafted the manuscript. All authors read and approved the final manuscript.

## Authors' information

*Jan J. van Wingerden*, MBChB, MMed (PlastSurg)(UP), FCS (SA) is a consultant reconstructive plastic surgeon with a special interest in complex thoracic wall- and, intrathoracic flap reconstructions and postpneumonectomy syndrome.

*Patrique Segers*, MD, PhD, is a senior registrar in cardiothoracic surgery. A substantial part of research for his PhD thesis was on topical negative-pressure (NPWT) in cardiothoracic cases.

*Lilian Jekel*, MD is an experienced cardiothoracic surgeon.

Further information on the authors can be obtained at http://www.ctsnet.org/
